# An analytical study on the awareness and practice relating toxoplasmosis among pregnant women in Casablanca, Morocco

**DOI:** 10.1186/s12889-021-10474-9

**Published:** 2021-03-16

**Authors:** S. Ait Hamou, M. Laboudi

**Affiliations:** 1 Laboratory of Ecology and Environment (LEE), Faculty of Sciences Ben M’Sik, Hassan II University, Casablanca, Morocco; 2 Department of Parasitology, National Institute of Hygiene, Rabat, Morocco

**Keywords:** Toxoplasmosis, Pregnant women, Knowledge, Practice, Casablanca, Morocco

## Abstract

**Background:**

Although toxoplasmosis is asymptomatic in most cases among pregnant women, it may cause newborn abortions and birth defects if the infection occurs during pregnancy. Therefore, raising awareness and promoting good practices of pregnant women towards toxoplasmosis disease is essential to avoid infection during pregnancy. The aim of this cross-sectional study is to assess toxoplasmosis awareness and its risk-related behavior pregnant women who attended public health centers in one of the biggest Moroccan cities: Casablanca.

**Methods:**

A structured questionnaire was used to collect data including socio-demographics data, awareness of etiology, modes of transmission and preventive practices towards toxoplasmosis.

**Results:**

This study survey showed that among 390 pregnant women interviewed, 41.2% reported having heard or read information regarding toxoplasmosis. Only 8.1% of them knew that toxoplasmosis is a parasitic disease caused by *Toxoplasma gondii,* and 13.7% of those who gave a correct answer were aware that the host of toxoplasmosis is cats*.* There is a significant statistical association between age, level of education and profession of pregnant women and toxoplasmosis awareness. Despite of the majority of the interviewed pregnant women had had effective preventive practices towards toxoplasmosis, 17.4% of pregnant women were convinced that Toxoplasmosis can transmit to the fetus and 14.3% stated it is asymptomatic in most cases.

**Conclusion:**

This study highlights the low level of knowledge and awareness of toxoplasmosis among pregnant women in Casablanca/Morocco. Therefore, a special education program targeting all women during their reproductive age is necessary.

## Introduction

Toxoplasmosis is a worldwide zoonotic infectious disease caused by *Toxoplasma gondii (T. gondii)* [1]. It is estimated that about a third of the World’s population is infected with toxoplasmosis [2]. Most *T. gondii* infections transmitted to humans are asymptomatic. However, up to 10% of infected people may present lymphadenopathy or eye disease [3]. The most severe symptoms concern seronegative women who are infected during pregnancy. *T. gondii* tachyzoites can be transmitted to the fetus [4]. The stage of pregnancy in which maternal toxoplasmosis occurs is an important factor in the frequency of transmission and severity of congenital infection. The risk of transmission is relatively low (< 20%) during the first trimester but increases to nearly 80% by the end of pregnancy. It is important to note that the consequences on the fetus are more severe when the infection occurs early in gestation [3, 5]. This congenitally acquired infection can have serious consequences such as abortion, stillbirth, neonatal death and central nervous system abnormalities at birth or ocular toxoplasmosis which affects the quality of life of the child throughout his lifetime [6]. However, clinical manifestations in individuals who have been congenitally infected may not be observed at birth, but later, in life [5].

Many serological surveys have reported seroprevalence rates of toxoplasmosis worldwide. They have also showed that a considerable variation in different parts of the world (from 7.5 to 95%) [7]. In Morocco, previous studies have estimated that approximately 50% of pregnant women were infected with *T. gondii* [8], with a risk factor related to their ignorance of the disease as well as their contact with the soil [9]. However, no studies on the prevalence of congenital toxoplasmosis have been reported to date. At present, the Moroccan health system does not have a monitoring program for toxoplasmosis and there is no national screening program for toxoplasmosis in the country. Therefore, lack of a systematic screening for this parasitosis to properly control the risk of infection of a congenital toxoplasmosis in our country means that there is no follow-up of pregnant women until delivery. A study conducted at the National Institute of Hygiene in Rabat showed that 28% of pregnant women are screened for toxoplasmosis for the first time with a pregnancy age of more than 5 months [10]. This leads us to question how far pregnant women, who had consulted Casablanca health centers, know about the disease. Nevertheless, several countries reported the assessment of pregnant women’s knowledge of toxoplasmosis [11, 12]. In 2003, Jones et al. reported that 48% of pregnant women have heard of toxoplasmosis but only 7% were aware of being tested for the disease [11]. In addition, Elsafi et al., [13] reported that 75.5% of pregnant women had never heard of toxoplasmosis. The authors concluded that the prevention of congenital infections should be a national priority and that all pregnant women should be informed about the risk of toxoplasmosis. It is, therefore, vital to provide a formal education about toxoplasmosis risk factors to women at childbearing age [12].

In Morocco, previous studies have reported that the disease remains neglected and scarcely documented in Morocco country [8], There are a very few studies which assessed the state of toxoplasmosis-related knowledge and practice among pregnant women. The aim of the present survey is to evaluate the awareness and preventives practice concerning toxoplasmosis among pregnant women attending primary health care in Casablanca in Morocco.

## Methods

### Study area

This study was carried out in Casablanca, the biggest city and the economic capital of Morocco. It is also the largest city in the Maghreb, as well as one of the largest and most important cities in Africa, both economically and demographically. Casablanca is located in the central-western part of Morocco bordering the Atlantic Ocean. According to the 2014 population estimate, the majority of the population (approximately 98%) lives in urban areas. The city has a very young population, with about one-quarter under the age of 15 years. The population, which exceeds 3 million, accounts for about 11% of the population of the entire country.

### Sampling and sample size

**Fig. 1:**
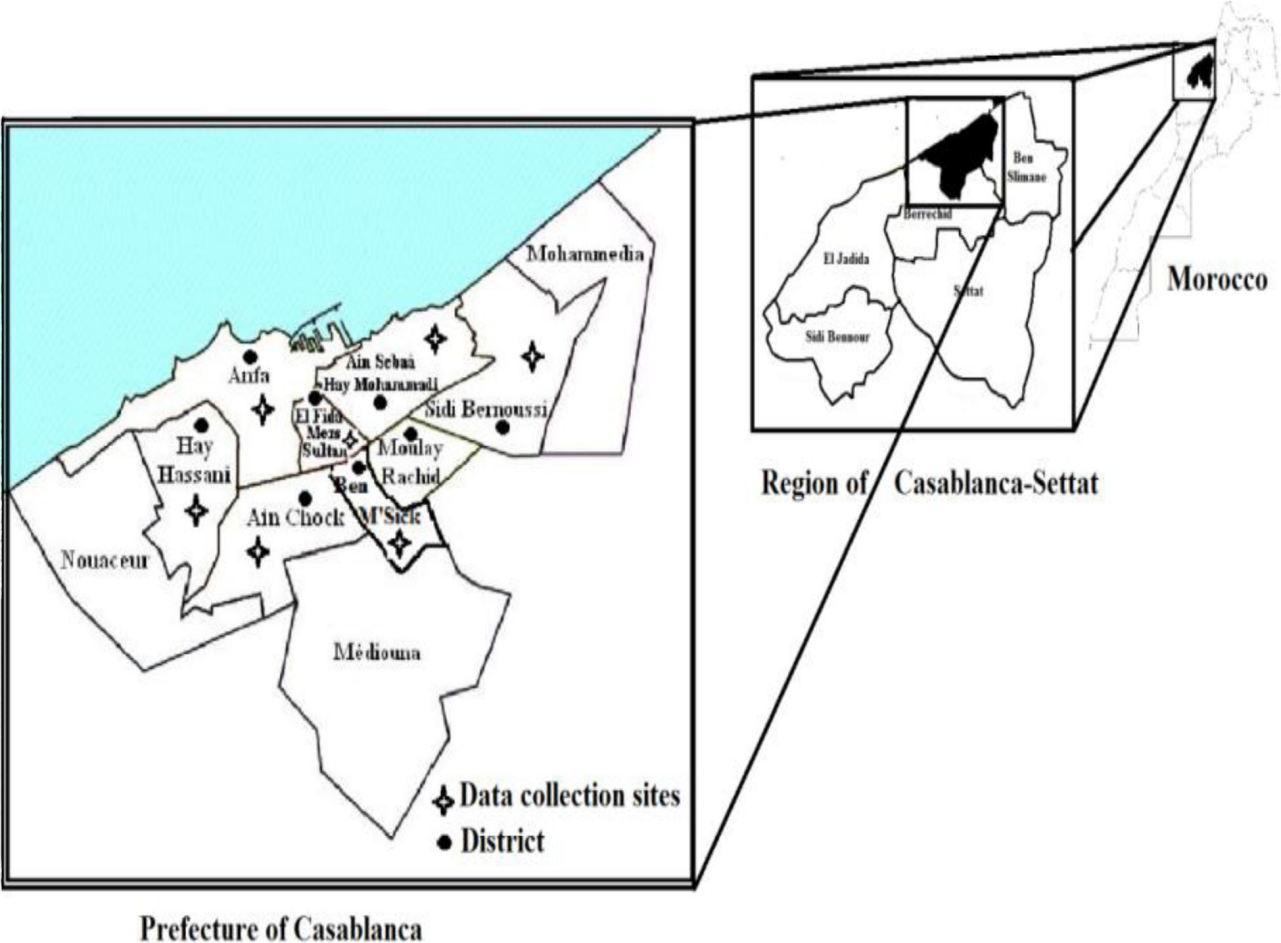
Area study

A cross-sectional study was carried out during the period of January to May 2018 in sixteen primary health centers in Casablanca. The centers were randomly chosen from an updated list in web portal of the Moroccan Ministry of Health (Fig. 1). As to the sample size, we used the WHO formula [13]. Using this formula of sample size, the minimum sample obtained as 384 with the prevalence was taken as 50%, the confidence level and absolute error of margin was a 95 and 5% respectively. During the study, the number of participants surveyed became 390. Informed consent was sought from all participants prior to enrolment in the study. The health centers were visited by the interviewers twice a week until the required sample size was reached. A convenience sample of pregnant women who were present on the day of data collection were included in the study. There were no special criteria for inclusion of the pregnant women. All the pregnant women who attended in these health facilities on the day of survey had the same chance of being included. Pilot testing was done in 2017 among pregnant women in health care center of Casablanca in Morocco.

### Data collection

A structured questionnaire designed according to the objectives of the study was used to assess toxoplasmosis’s general knowledge and its preventive practices. The questionnaire was estimated to take about 20 min. The translation of the questionnaire in to Arabic helped the face-to-face interviews conducted by the investigators, who briefed the participants about the study objectives and provided guidance. The questions were answered orally by the interviewees and recorded by the investigators in order to facilitate the understanding of the subject matter. The first part of the questionnaire is dedicated to socio-demographic characteristics such as: age, residence, level of education, gestational age, parity and abortion history. The second part questionnaire focused on knowledge of the disease, which included general information about the causative agent, the definitive host of the disease, diagnosis, risk factors, symptoms and signs. The last part of the questionnaire enquired about toxoplasmosis’s preventive practices such as contact with cats, consumption of undercooked meat, consumption of unwashed fruits and vegetables, drinking of water (treated or untreated), the regular washing of hand’s after contacting raw meat and before eating.

### Data analysis

All the data from the questionnaire was put together in Epiinfo software 2012 *version (3.5.4).* Descriptive statistics using frequencies and percentages were used to identify participants’ knowledge about toxoplasmosis. A chi-square (*X*^2^) test was performed to examine the association among the categorical variables such as the relationships between the different characteristics of participants with some variables included in the questionnaire. The results will be considered statistically significant when *p < 0.05*.

## Results

### Socio-demographic characteristics of pregnant women

A total of 390 pregnant women accepted to participate in the study. The mean age of the participants was 29.7 ± 6.8 years. 67.4% of respondents have an urban background. 24.9% of the interviewees are illiterate while 20.5% of them had completed their high school education. Most respondents were in the first trimester of their pregnancy (41.5%), 36.9% in the second trimester, while the remaining 21.6% were in their third trimester (Table 1). Among the pregnant women participated, 27.9% of the participants in the study claimed to have an abortion history. Furthermore, the majority of pregnant women were housewives (91.3%) and 54.9% of them had more than three to four pregnancies.

**Table 1:** Socio-demographic characteristics of pregnant women consulting in public health facilities in Casablanca in Morocco

	N	%	95% CI	
**Age (Years)**				
< 25 years	96	24.7%	20.6%	29.4%
25–34 years	193	49.7%	44.7%	54.8%
≥ 35 years	99	25.6%	21.3%	30.2%
**Parity**				
Nulliparous (=0)	136	34.9%	30.2%	39.9%
Pauciparous(1–2)	214	54.9%	49.8%	59.9%
Multiparous (≥ 3)	40	10.2%	7.5%	13.8%
**Residence**				
urban	263	67.4%	62.5%	72.0%
suburban	15	3.8%	2.2%	6.4%
rural	112	28.8%	24.3%	33.5%
**Gestational age**				
First trimester	162	41.5%	36.6%	46.6%
Second trimester	144	36.9%	32.2%	41.9%
Third trimester	84	21.6%	17.6%	26.0%
**Educational level**				
Illiterate	97	24.9%	20.7%	29.5%
Incomplete elementary school	137	35.1%	30.4%	40.1%
incomplete high school	76	19.5%	15.7%	23.8%
Complete higher education	80	20.5%	16.7%	24.9%
**History of abortion**				
Yes	109	27.9%	23.6%	32.7%
No	281	72.1%	67.3%	76.4%
**Profession**				
Housse wife	356	91.3%	88.0%	93.9%
Working	34	8.7%	6.2%	12.1%

### Toxoplasmosis related awareness and sociodemographic characteristics

To identify potential associations between toxoplasmosis awareness and socio-demographic characteristics, a chi-square (*X*^2^) test was conducted (Table 2). There was a significant difference between the knowledge of toxoplasmosis and the age, level of education and profession. The chance of toxoplasmosis-related knowledge was higher among women aged 25 to 34 years old with 47.2% than to the 17 to 24 years old age group as only 31.3% of them had toxoplasmosis-related knowledge. 68.8% of them had a higher level of education; they had an awareness of the disease, heard or read about toxoplasmosis; while 32.1% had not finished their elementary school education (*p < 0.05*). As to the socio-professional milieu that the respondents belonged to, 79.4% of the pregnant women who had a job were highly aware of the disease; while (37.6%) (*p < 0.05*) who were housewives had a lesser awareness. On the other hand, it is worth noting that no significant association was found between parity, residence, history of abortion and the gestational age and toxoplasmosis awareness (*p > 0.05*).

**Table 2:** Distribution of pregnant women treated in public healthcare service according to age, gestational age, parity, history of abortion, profession and toxoplasmosis aware (heard/read) in Casablanca in Morocco (*N* = 390)

	Heard/read of toxoplasmosis n (%)	Never heard/read of toxoplasmosis n (%)	***N***	***X***^***2***^	***p***
**Age (Years)**					
< 25	30(31.2)	66(68.8)	96	6.87	0.0321*
25–34	92(47.2)	102(52.8)	193		
35–48	39(39.4)	60(60.6)	99		
**Parity (number of pregnancy)**					
Nulliparous (=0)	50(36.8)	86(63.2)	136	4.21	0.1215
Pauciparous (1–2)	98(45.8)	116(54.2)	214		
Multiparous (≥ 3)	13(32.5)	27(67.5)	40		
**Residence**					
Urban	116(44.1)	147(55.9)	263	2.68	0.2607
Suburban	5(33.3)	10(66.7)	15		
Rural	40(35.8)	72(64.2)	112		
**Gestational age**					
First trimester	63(38.9)	99(61.1)	162	1.83	0.3997
Second trimester	58(40.3)	86(59.7)	144		
Third trimester	40(47.6)	44(52.4)	84		
**Educational level**					
Illiterate	29(29.9)	68(70.1)	97	34.97	0.0000*
Incomplete elementary school	44(32.1)	93(67.9)	137		
Incomplete high school	33(43.4)	43(56.6)	76		
Complete higher education	55(68.8)	25(31.2)	80		
**History of abortion**					
Yes	42(38.5)	67(61.5)	109	0.47	0.4920
No	119(42.3)	162(57.7)	281		
**Profession**					
Houssewife	134 (37.6)	222(62.4)	356	22.34	0.0000*
Working	27(79.4)	7(20.6)	34		

*statistically significant (*p* < 0.05)

### Respondents’ awareness of toxoplasmosis

Most of the surveyed women were unaware (58.8%) (229/390) [53.6–63.6%] of the existence of toxoplasmosis. They claimed to have never heard or read information about it. Only 41‚2% (161/390) [36.4–46.4%] were aware of the disease (Table 3). Of the 161 women, who reported to have acquired knowledge about toxoplasmosis from different sources. 62.2% claimed to have heard about toxoplasmosis from the family, 15% said it is from the media; while 11.7% said they got information from health professionals. It is also worth observing that 91.9% of the respondents were unable to identify the etiologic agent of toxoplasmosis (Table 3); and 13.7% of women associated the parasite with cats. Furthermore, only 17.4% were convinced that Toxoplasmosis can transmit to the fetus when the pregnant woman contracts the parasite during pregnancy. Additionally, 14.3% stated that toxoplasmosis is an asymptomatic disease in most cases. As far as the diagnosis and seroconversion of toxoplasmosis is concerned, more than 90% of the interviewees did not have any idea about these subjects. On the other hand, more than half of them were certain about the ways to contract the disease: 71.4% acknowledged that Toxoplasmosis contracts mainly through food (Table 3).

**Table 3:** Responses of pregnant women on awareness of the epidemiology, clinical and risk factors associated with exposure to toxoplasmosis (*n* = 161)

General information toxoplasmosis knowledge	n	***%***	***95% CI***
Have you ever read or heard of toxoplasmosis?			
Yes	161	41‚2%	36.4–46.4%
No	229	58.8%	53.6–63.6%
Toxoplasmosis is parasite disease caused by Toxoplasma gondii?			
Yes^a^	13	8.1%	4.4–13.4%
No	4	2.5%	0.7–6.2%
Don’t know	144	89.4%	83.6–93.7%
Is the host of toxoplasmosis is the cat?			
Yes^a^	22	13.7%	8.8–20.0%
No	129	80.1%	73.1–86.0%
Don’t know	10	6.2%	3.0–11.1%
Can pregnant women develop serious complications after toxoplasmosis infection?			
Yes^a^	67	41.6%	33.9–49.6%
No	5	3.1%	1.0–7.1%
Don’t know	89	55.3%	47.3–63.1%
Do you know the seroconversion of toxoplasmosis?			
Yes	7	4.3%	1.8–8.8%
No	154	95.7%	91.2–98.2%
Toxoplasmosis in pregnant women is asymptomatic in most cases?			
Yes^a^	23	14.3%	9.3–20.7%
No	10	6.2%	3.0–11.1%
Don’t know	128	79.5%	72.4–85.5%
Can toxoplasmosis transmit from pregnant woman to her fetus if she is newly infected during pregnancy?			
Yes^a^	28	17.4%	11.9–24.1%
No	4	2.5%	0.7–6.2%
Don’t know	129	80.1%	73.1–86.0%
Do you know the diagnosis of toxoplasmosis?			
Yes	5	3.1%	1.0–7.1%
No	156	96.9%	92.9–99.0%
**Risk factors**			
Consumption of fruit or vegetable with faces			
Yes^a^	110	68.3%	60.5–75.4%
No	14	8.7%	4.8–14.2%
Don’t know	37	23%	16.7–30.3%
Consumption of untreated water			
Yes^a^	113	70.2%	62.5–77.1%
No	15	9.3%	5.3–14.9%
Don’t know	33	20.5%	14.5–27.6%
Contact direct with faces of cat			
Yes^a^	111	68.9%	61.2–76.0%
No	14	8.7%	4.8–14.2%
Don’t know	36	22.4%	16.2–29.6%
Eating undercooked meat			
Yes^a^	114	70.8%	63.1–77.7%
No	22	13.7%	8.8–20.0%
Don’t know	25	15.5%	10.3–22.1%
Toxoplasmosis contracts mainly through food?			
Yes^a^	115	71.4%	63.8–78.3%
No	23	14.3%	9.3–20.7%
Don’t know	23	14.3%	9.3–20.7%

^a^The correct answer

### Toxoplasmosis’s practices

Approximatively, 95% of respondents indicated that they routinely wash their hands after handling meat or before eating, thoroughly washed fruits and vegetables before eating and use treated water for drinking (Table 4).

**Table 4:** Practice of pregnant women towards toxoplasmosis (*n* = 390)

	n	***%***	***95% CI***
**Contact with cat**			
Yes	36	(9.23%)	87.4–93.5%
No	354	(90.77%)	6.6–12.7%
**Consumption of undercooked meat**			
Yes	22	(5.64%)	91.5–96.3%
No	368	(94.36%)	3.7–8.5%
**Drinking treated water**			
Yes	372	(95.38%)	92.7–97.2%
No	18	(4.62%)	2.8–7.3%
**Washing fruit and vegetable before consumption**			
Yes	373	(95.64%)	93.0–97.4%
No	17	(4.36%)	2.6–7.0%
**Washing hands after handling meat**			
Yes	373	(95.64%)	93.0–97.4%
No	17	(4.36%)	2.6–7.0%
**Regular hand washing before eating**			
Yes	374	95.9%	93.3–97.6%
No	16	4.1%	2.4–6.7%

## Discussion

Primary prevention of toxoplasmosis seeks to strengthen pregnant women’s awareness related to the causative agent, route of transmission. The above mentioned healthy measures may reduce toxoplasmosis’s potentially tragic outcome for both the fetus and the newborn [14]. To our knowledge, this is the first study which was conducted in Casablanca to assess the awareness level and to highlight the best practices towards toxoplasmosis among pregnant women. Indeed, the present study has demonstrated that the majority of pregnant women were unaware of toxoplasmosis and only 41.3% of them had heard or read about toxoplasmosis. Previous studies in others part of the world reported low toxoplasmosis awareness. In Egypt, It was found that only 9.9% of the studied sample had a good knowledge of toxoplasmosis [15]. Similar results were reported in Tanzania and in Ethiopia, where only 5 and 5.7%, respectively, of pregnant women had knew about the disease [16, 17]. Our findings are closer to the ones reported in Brazil, where (44%) of pregnant women claimed to know toxoplasmosis [18]*.* The low knowledge of toxoplasmosis (11%) was also reported in Asian country such as Malaysia, Philippines and Thailand [19]. Besides, in the United States (USA), Jones et al.*,* (2003), who assessed toxoplasmosis-related knowledge and best practices among pregnant women, revealed that less than half of their sample heard or read information about toxoplasmosis [11]. By contrast, in Iraq, 64.7% of pregnant women had heard of the disease [20]. This rate difference may be due the different cultural or socio-demographic factors in each country.

The low awareness of pregnant women obtained in our survey is probably due to the absence of toxoplasmosis screening program in Morocco. This could be the reason behind this lack of awareness and knowledge about toxoplasmosis. A previous study in Poland has highlighted the role of prevention programs on the incidence of seroconversions between 1991 and 1997. The authors have concluded that the knowledge of pregnant women has almost doubled from 24.3% in 1991 to 45.3% in 1997 [21]*.*

Obviously, not knowing the disease has made 58.7% of pregnant women with no toxoplasmosis awareness to be part of a high-risk group. They are likely to be infected with *T. gondii* infection during pregnancy, which might consequently lead to an acute infection and raise the risks of congenital transmission. Elsafi et al.*,* (2015) observed that pregnant women’s unawareness of the disease could significantly increase the risk of Toxoplasmosis infection [12]. Indeed, healthcare professionals should advise patients that the only tool to avoid *Toxoplasma* seroconversion during pregnancy is through primary prevention. They must provide general information on the infection, the parasite, surveillance during pregnancy and disseminate preventive hygiene and diet recommendations. Unfortunately, some health professional including physicians and nurses had a modest knowledge on this parasitic infection; they have thus failed to provide sufficient information to pregnant women [22]. Therefore, in our country, health professionals should constantly update their information about toxoplasmosis and need to deepen their understanding of toxoplasmosis, and the practices needed to avoid it. Alternatively, an appropriate health education could be provided to pregnant women during pregnancy.

The present study has demonstrated that there is a positive correlation between women’s awareness and their age, level of education and profession. This understanding of the disease was significantly higher among women whose age range from 35 to 44 years old, and who have an academic education. Similar findings were reported in Egypt. There, researchers recorded a positive correlation between women’s awareness of the disease and their age and level of education [15]. These findings are in turn similar with those reported from studies in the USA where pregnant women aged 25 to 34 years with high education were more likely to have a prior knowledge of *T. gondii* [11, 23]. Furthermore, as has been demonstrated in our study, the profession seems to be significantly associated with toxoplasmosis awareness. However, the present results contradicts with the study of Moura et al.*,* (2019) who found out that there is a negative correlation between the occupation and the knowledge of pregnant women [18]*.* Education is important for pregnant women as it increases their awareness as to the importance of hygiene to prevent all sorts of diseases including toxoplasmosis. Hence, the educational level can be a protection factor against *T. gondii* infection during pregnancy [20, 24].

In general, the present study has revealed that women had a poor knowledge of toxoplasmosis, its causative agent and its definitive host disease, cats. In Essaouira, only 2.66% of pregnant women who have had the appropriate information about the mode of transmission and its complications in both the fetus and the mother [25]. The study carried out in Iraq has demonstrated that the high rate of individuals who failed to provide the appropriate answer the causative agent of the disease [20]. By contrast, previous studies carried out in Egypt, USA and Asia have observed that pregnant women have a deeper understanding about the causative infection and that cats are definitive host of disease [11, 15].

A better understanding of toxoplasmosis’s routes of transmission is crucial for the prevention of infection among pregnant women. In this survey, we have noted reported that more than half the respondents knew about the route of the disease’s transmission such as eating undercooked meat, contact with cats, consumption of untreated water and fruit or vegetable as a risk factor for infection. These findings remain, however, insufficient to reduce the risk of seroconversion during pregnancy. It was corroborated with study in the USA, where the majority of the pregnant women interviewed were aware of the appropriate preventive measures to avoid infection [11]. By contrast, in Ethiopia, most of the respondent pregnant women were not sure about the risk factors, symptoms, and timing of infection of toxoplasmosis [17]. A low proportion (45.3%) of pregnant women with a good knowledge of the risk factors for contamination of toxoplasmosis was reported in the Polish study [21]*.* The same findings recorded in Brazil where only 23.4% of the pregnant women had a deeper knowledge of the disease, mainly in the area of prevention, while 58.9% adopted suitable preventive behaviors [18]. In this regard, an appropriate knowledge of toxoplasmosis’s risk factors among pregnant women boosts preventive behaviors against severe the complications that result from congenital infection, and that it is only this knowledge that enables pregnant women to reduce the risk of fetal infection [21, 26].

The study has come up with an unexpected finding: the majority of the interviewed pregnant women had a positive attitude towards the preparation and cooking of meat, the washing their hands after handling meat, the washing of fruits and vegetables before eating and the use of treated water for drinking, consumption of undercooked/partially cooked meat and contact with cats. Probably, the pregnant women under the present study were properly avoided the risk behaviors, without realizing what they were avoiding. The same findings were was reported in Egypt (2014), where the majority of the studied women had a positive attitude towards the preparation and cooking of meat, importance of the use of soap while washing hands after handling meat, vegetables, in addition to avoid playing with cats [15]*.* In Tanzania, Onduru et al.*,* [16] recorded that preventive practices towards toxoplasmosis among pregnant women could also be due to improved sanitation behaviors and standards of living. The authors reported that the majority of women did not eat raw or undercooked meat and 58% drank untreated water which could harbor the parasite oocysts and serve as a potential source of *Toxoplasma* infection [16]*.* Similar results were reported in France in 1999, which concluded that the riskiest behaviors were poor hand hygiene, the eating of undercooked meat, cats’ possession, frequent consumption of raw fruits and vegetables outside home, as well as the consumption of raw vegetables badly washed or contaminated with kitchen utensils [27]*.* From Egypte, 84.1% had a positive attitude towards toxoplasmosis and 15.9% had a negative one [15]. In Brazil, on the other hand, 58.9% of pregnant women adopted a preventive toxoplasmosis behavior, and 41,1% did not present it [18]. Our findings reiterate the importance of health education and toxoplasmosis awareness in decreasing the incidence of the disease and the burden of the effects of congenital toxoplasmosis. Health education in infectious diseases is essential during pregnancy. Such an education can be carried through campaigns, lectures and other educational programs to teach women the essential recommendations to follow to avoid contact with the disease.

### Limitation of study

The study has some limitations. Firstly, the study might not be able to represent the knowledge and perception of this parasitic in the large population of pregnant women in Casablanca region. Secondly, no serological analysis of interviewed pregnant women was done to complement the questionnaire survey.

## Conclusion

This is the first report assessing Toxoplasmosis knowledge and practice among pregnant women in Casablanca city in Morocco. Most pregnant women participated in our study were unaware of toxoplasmosis infection, therefore, It is crucial to improve toxoplasmosis information to pregnant women during pregnancy.it emphasizes the need for implementation of program surveillance of toxoplasmosis by Ministry of Health in Morocco which involved health education to ensure reproductive health to the pregnant women about disease in order to prevent the risks for the fetus and consequently to reduce the incidence of congenital toxoplasmosis.

In addition, update the knowledge for medical personnel, is also recommended. This can be done through strengthening of the curriculum for training of enrolled nurses and midwives to cover more aspects of congenitally transmitted diseases including toxoplasmosis.

## Data Availability

The datasets used during the current study are available from the corresponding author.

## References

[1] Petersen E, Vesco G, Villari S, Buffolano W. What do we know about risk factors for infection in humans with *Toxoplasma gondii* and how can we prevent infections? Zoonoses Public Health. 2010;57(1):8–17. 10.1111/j.1863-2378.2009.01278.x 19744301

[2] Pappas G, Roussos N, Falagas ME (2009). Toxoplasmosis snapshots: global status of *Toxoplasma gondii* seroprevalence and implications for pregnancy and congenital toxoplasmosis. Int J Parasitol.

[3] Montoya JG, Liesenfeld O (2004). Toxoplasmosis. Lancet.

[4] Montoya JG, Remington JS. Clinical practice: Management of *Toxoplasma gondii* infection during pregnancy. Clin Infect Dis. 2008;47(4):554–66. 10.1086/590149 18624630

[5] Rorman E, Zamir CS, Rilkis IHB. Congenital toxoplasmosis-prenatal aspects of *Toxoplasma gondii *infection. Reprod Toxicol. 2006;21(4):458–72. 10.1016/j.reprotox.2005.10.006 16311017

[6] Tenter AM, Heckeroth AR, Weiss LM (2000). *Toxoplasma gondii*: from animals to humans. Int J Parasitol.

[7] Asthana SP, Macpherson CN, Weiss SH, Stephens R, Denny TN, Sharma RN, Dubey JP. Seroprevalence of *Toxoplasma gondii* in pregnant women and cats in Grenada, West Indies. J Parasitol. 2006;92(3):644–5. 10.1645/GE-762R.1 16884013

[8] Laboudi M. Review of toxoplasmosis in Morocco: Seroprevalence and risk factors for *T**oxoplasma* infection among pregnant women and HIV- infected patients. Pan Afr Med J. 2017;27(269):1–6. 10.11604/pamj.2017.27.269.11822 PMC5660321 29187938

[9] Laboudi M, El Mansouri B, Sebti F, Amarir F, Coppitiers Y, Rhajaoui M (2009). Facteurs de risque d’une sérologie toxoplasmique positive chez la femme enceinte au Maroc. Parasite..

[10] Laboudi M. Sérodiagnostic de la toxoplasmose chez les femmes enceintes à l’Institut National d’Hygiène de Rabat, 3rd International meeting on Clinical Infectious Diseases” sous le thème: « Congenital Toxoplosmosis Infection at Birth: An Update on pathology. In: Oral communication. 2016.

[11] Jones JL, Ogunmodede F, Scheftel J, Kirkland E, Lopez A, Schulkin J, Lynfield R. Toxoplasmosis-related knowledge and practices among pregnant women in the United States. Infect Dis Obs Gynecol. 2003;11(3):139–45. 10.1080/10647440300025512 PMC1852280 15022874

[12] Elsafi SH, Al-Mutairi WF. Al-Jubran, KM, Abu Hassan MM, Al Zahrani E. Toxoplasmosis seroprevalence in relation to knowledge and practice among pregnant women in Dhahran, Saudi Arabia. Pathog Glob Health. 2015;109(8):377–82. 10.1080/20477724.2015.1103502 PMC4809232 26924348

[13] World Health Organization(WHO). Adequacy of Sample Size in Health Studies. 1991.

[14] Ross DS, Jones JL, Lynch MF. Toxoplasmosis, cytomegalovirus, listeriosis, and preconception care. Matern Child Health J. 2006;10(1):189–193. 10.1007/s10995-006-0092-0. 10.1007/s10995-006-0092-0 PMC1592156 16752091

[15] Abdalla Sayed Ahmed GM, Abo Elghite Elhossiny EE. Knowledge and Attitude of women regarding Toxoplasmosis during pregnancy and measures to overcome it in Slums areas. Int J Curr Res.2014;6(4):6365–71.

[16] Onduru OG, Fred-Rumisha S, Munyeme M. Phiri AM. Evaluation of the level of awareness of congenital toxoplasmosis and associated practices among pregnant women and health workers in tanzania’s temeke district in dar es Salaam. Afr Health Sci. 2019; 19(4):3027–37. 10.4314/ahs.v19i4.24 PMC7040330 32127878

[17] Hdush Desta A. Knowledge, Attitude and Practice of community towards zoonotic importance of Toxoplasma infection in Central Afar Region, North East Ethiopia. Int J Biomed Sci Eng. 2015;3(6):74-86.

[18] Moura IPSI, Ferreira IP, Pontes AN, Bichara CNC (2019). Toxoplasmosis knowledge and preventive behavior among pregnant women in the city of Imperatriz, Maranhão, Brazil. Cien Saude Colet.

[19] Andiappan H, Nissapatorn V, Sawangjaroen N, Khaing SL, Salibay CC, Cheung MMM, Dungca JZ, Chemoh W, Teng CX, Lau YL, Mat Adenan NA. Knowledge and practice on Toxoplasma infection in pregnant women from Malaysia, Philippines, and Thailand. Front Microbiol. 2014;5(JUN):1–8. 10.3389/fmicb.2014.00291 PMC4052801 24966855

[20] Obaid HM. Toxoplasma sero-prevalence and related knowledge survey in pregnant women and university staff. Int J Curr Microbiol Appl Sci. 2019;8(02):2808–16.

[21] Pawlowski ZS, Skommer J, Paul M, Rokossowski H, Suchocka E, Schantz P. Impact of health education on knowledge and prevention behavior for congenital toxoplasmosis: the experience in Poznan, Poland. Health Educ Res. 2001;16(4):493–502. 10.1093/her/16.4.493 11525395

[22] Laboudi M, AitHamou S, Mansour I, Hilmi I SA. The first report of the evaluation of the knowledge regarding toxoplasmosis among health professionals in public health centers in Rabat, Morocco. Trop Med Health. 2020;48(17):1–8 10.1186/s41182-020-00208-9 PMC7144052 32292287

[23] Ogunmodede F, Scheftel J, Jones, JL. Lynfield R. Toxoplasmosis prevention knowledge among pregnant women in Minnesota. Minn Med. 2005;88(2):32–4. 17886796

[24] Millar PR, Moura FLD, Bastos OMP, Mattos DPBG FA, Sudré AP, Leles D AM. Conhecimento sobre toxoplasmose entre gestantes e puérperas atendidas na rede pública de saúde do município de Niterói, Rio de Janeiro, Brasil. Rev Inst Med Trop Sao Paulo. 2014;56(5):433–8. 10.1590/S0036-46652014000500011 PMC4172116 25229225

[25] Ouzennou N, Boussaa S, Ben Alla SBA. Observational study to assess pregnant women’s knowledge and behaviour related to toxoplasmosis in Essaouira province, Morocco. Asian Pac J Trop Med. 2019;12(2):87–90.

[26] Carter A, Gelmon SB, Toepell AP. The effectiveness of a prenatal education programme for the prevention of congenital toxoplasmosis. Epidemiol Infect. 1989;103(3):539–45. 10.1017/s0950268800030934 PMC2249529 2606162

[27] Baril L, Ancelle T, Goulet V, Thulliez P, Tirard-Fleury V, Carme B (1999). Risk factors for toxoplasma infection in pregnancy: a case-control study in France. Scand J Infect Dis.

